# Possible Association between Physical and Cognitive Function and Stumbling and Falling in Elderly Workers

**DOI:** 10.3390/ijerph192113826

**Published:** 2022-10-24

**Authors:** Kotaro Morita, Kazuhiro Nogawa, Yuuka Watanabe, Sayaka Sakuma, Koichi Sakata, Katsuyuki Ito, Chika Kumeda, Yasushi Suwazono

**Affiliations:** Department of Occupational and Environmental Medicine, Graduate School of Medicine, Chiba University, Chiba 260-8670, Japan

**Keywords:** elderly workers, stumbling, fall injury, physical function, cognitive function

## Abstract

The aim of this paper is to examine the association between physical and cognitive function and stumbling and falling in elderly workers by conducting work-related questionnaire surveys and physical and cognitive function measurements. A total of 611 men and 121 women aged 40–69 years who participated in physical function measurements between June 2017 and June 2021 were included in the study. The general physical function measurements of upper and lower limb muscle strength, dynamic and static balance, and agility and cognitive function included grip strength, Repeated Rise Test, Trail Making test (TMT), and Three-Meter Time Up Go Test (TUG). We also asked the men and women about their experience of falling and stumbling. Logistic regression analysis showed significant odds ratios (OR) for the associations between stumbling in men and age (OR: 1.98), mental burden (OR: 2.44), frequency of field work (OR: 1.74), seated stepping test count (OR: 0.95), and TMTB time (OR: 0.99). Significant ORs were found between falling in men and age (OR: 2.55), mental burden (OR: 2.40), exercise habits (OR: 2.55), and smoking (OR: 2.00). Significant ORs were found between stumbling in women and d_TUG (OR: 1.59) and mental burden (OR: 6.42). The study suggests that there may be an association between cognitive and physical decline and stumbling and falling in elderly workers.

## 1. Introduction

Japan’s population is aging at a rate that is unprecedented in the world. It is estimated that by 2065, the aging rate will reach 38.4%, with 1 out of every 2.6 people in Japan aged 65 or older [1]. To maintain the vitality of the economy and society in the midst of a rapidly declining birthrate and aging population, the Law Concerning Stabilization of Employment of Older Persons was partially revised and came into effect on 1 April 2021, with the aim of creating an environment where older people who are willing to work can fully demonstrate their abilities. Compared with the elderly in other countries, a greater proportion (40.2%) of the Japanese elderly desire to work (or continue to work) with income, indicating that they are highly motivated to work [1]. As the number of older workers is increasing, occupational health activities for older workers are becoming more important. However, there is concern that in addition to the increase in cerebrovascular, cardiac, and other diseases due to aging, the decline in physical and cognitive functions among older workers is leading to occupational accidents. In fact, the number of occupational accidents in recent years has been highest among workers aged 60 and over, with 34,928 fatalities and injuries resulting in 4 or more days of absence from work in 2020 [2]. Fatalities and injuries to older workers account for 26.6% of all reported injuries and the highest number of accidents in the industry [2].

Regarding the decline in physical and cognitive functions of older adults, a comparison of the functional level of people aged 55–59 with people aged 20–24 shows that not only sensory functions (vision, hearing, etc.) and physical functions (balance, etc.) but also cognitive functions such, as learning ability and memory, have declined significantly with age [3,4]. However, because the subjects were under the age of 60 and because recent studies are not available, it is not yet clear to what extent physical and cognitive functions deteriorate in workers aged 60 and over.

According to this background, the aim of this study was to examine the factors associated with falls and stumbles in elderly workers by conducting work-related questionnaire surveys and physical and cognitive function measurements. We also aimed to provide information for a comprehensive evaluation index for the work ability of elderly workers.

## 2. Materials and Methods

**Figure 1:**
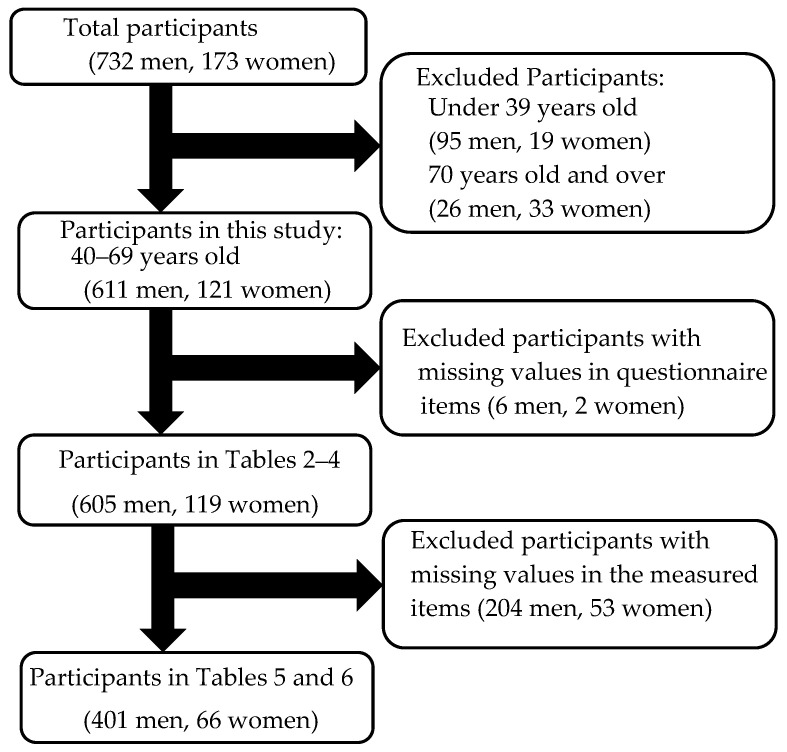
Participant flow diagram.

This study focused on older workers. Of a total of 905 participants (732 men and 173 women) who participated in physical function measurements carried out at each randomly selected company’s site between June 2017 and June 2021, 611 men and 121 women aged 40–69 were included. Figure 1 shows the participant flow diagram.

Physical function measures included grip strength, Repeated Rise Test, Seated Stepping Test, Closed-Eye One-Leg Test, Functional Reach Test (FRT), Trail Making Test A and B (hereafter TMTA and TMTB), 3 m Time Up Go Test (hereafter TUG), and TUG under a double task (hereafter d_TUG). In addition to the general physical function measurement items, such as upper and lower limb muscle strength, dynamic and static balance, and agility, we selected measures of cognitive function that are referred to widely in reports of stumbling and falling among elderly people [5,6,7,8,9]. Procedures for measuring physical function were as follows. Grip strength is a measure of upper limb muscle strength. A digital grip strength meter manufactured by Takei Scientific Instruments (Niigata, Japan) was used for these measurements. The participants, while in the standing position, grasped the grip strength meter downwards and carried out a total of four measurements, two each on the left and right sides, performed alternately. The measurements were performed without the grip strength meter touching the trunk, with the average value used in the analyses. The Repeated Rise Test measures the number of times a person can repeat a sitting and standing position in 30 s and is a measure of lower limb muscle strength. The TUG is a test that measures the time taken to stand up from a sitting position in a chair, go around a pole 3 m away, and sit down again in a chair, and is a measure of walking ability. The Seated Stepping Test measures the number of times a person can open and close their legs in 20 s while sitting in a chair, and is a measure of agility. The Closed-Eye One-Leg Test measures the number of seconds a person can hold a one-leg stand with closed eyes and is a measure of static balance. The Closed-Eye One-Leg Test was measured on each leg, left and right, and a longer time was used. The FRT is a test of dynamic balance, measuring how many centimeters are required to reach the maximum position at which balance can be maintained by leaning forward from an upright position with hands extended in front of the body. The FRT was measured twice, and the maximum value was used. In a previous study of 493 subjects aged 65 years or older who were examined using physical performance indices [5], the TMTA and TMTB were selected as measures of cognitive function, since the TMT reflects complex gait performance and could be a useful overall index in health programs to promote independence among Japanese elderly people. Each of the measures was selected to be as general and simple as possible so that they can be implemented by employees in the field, even in workplaces where no medical professionals are assigned.

Procedures for measuring cognitive function were as follows. The TMTA is a test that measures the time taken to connect numbers randomly listed from “1” to “25” with a line starting from “1,” and evaluates cognitive functions, such as attention, memory, and processing speed. The TMTB is a test that measures the time required to connect randomly listed numbers from “1” to “13” and hiragana from “あ” to “し” in alternating lines, such as “1” → “あ” → “2” → “い” → … → “12” → “し” → “13”, and measures cognitive functions reflecting higher-level information processing abilities. The d_TUG is a test in which the subject carries out a simple calculation task while walking, and is a measure of cognitive function. Each questionnaire item was classified as shown in Table 1. The main members of the team who performed the measurements were fixed at the coauthors, and the person in charge of the health checkup was sometimes in charge of the measurer, except for the coauthor. However, the same measuring instruments were used, with the measurements performed according to a manual in order to avoid large measurement errors for individuals performing the measurements. Since an actual stumbling or falling accident was considered to be rather rare, we adopted recent subjective susceptibility of stumbling or falling as the indicator in the present study.

### Statistics

A Mann–Whitney U test was performed on the continuous variables of the measurement items: age, grip strength, Repeated Rise Test, Closed-Eye One-Leg Test, FRT, Seated Stepping Test, TMTA, TMTB, TUG, and d_TUG. In addition, χ-square tests were conducted on the categorical variables of the questionnaire items: frequency of field work, physical burden, mental burden, low back pain, knee pain, exercise habits, smoking, frequency of drinking, and type of occupation. Of the 611 subjects, 401 (excluding those with missing values) were included in the analysis. Logistic regression analysis was conducted with “stumbling” and “falling” as the dependent variables, and age (+10 years), grip strength, Repeated Rise Test, Closed-Eye One-Leg Test, FRT, Seated Stepping Test, TMTA, TMTB, TUG, d_TUG, frequency of field work, physical burden, mental burden, low back pain, knee pain, exercise habits, smoking, frequency of drinking, and type of occupation as the independent variables, selected by backward elimination methods based on Wald statistics. All analyses were performed using the IBM SPSS 19J statistical software (IBM Business Analytics, Tokyo, Japan). *p*-Values < 0.05 were considered statistically significant.

## 3. Results

Excluding those with missing values in the questionnaire items, 605 men and 119 women were included, 202 (33.4%) in the yes-stumbling group, 84 (13.9%) in the yes-falling group, and 6 (1.0%) in the yes-getting stuck or entangled group. We also found that about 11% of the participants reported that they had both stumbled and fallen, indicating a certain number of overlapping risks (Table 2). There was little indication of subjects being stuck or entangled, and it was clear that there were few accidents at the subjects’ work sites. Therefore, the present study was designed to further examine the statistics of stumbling and falling. The general demographics of the participants are shown in Table 2 and the “Total” column in Table 3 and Table 4.

First, A Mann–Whitney U test was conducted on the continuous variables. In men, the yes-stumbling group was significantly older and performed significantly fewer Repeated Rise Tests and Seated Stepping Tests and a significantly shorter duration of the Closed-Eye One-Leg Test and a significantly longer duration of TMTA, TMTB, TUG, and d TUG. In women, the yes-stumbling group had a significantly longer duration of TUG and d TUG. Furthermore, in men, the yes-falling group was significantly older and had significantly lower grip strength, a significantly shorter duration of the Closed-Eye One-Leg Test, significantly lower counts of the Seated Stepping Test, and a significantly longer duration of TMTA, TMTB, and TUG. In women, the yes-falling group had a significantly longer duration of TUG [Table 3].

Next, a χ-square test was conducted on the categorical variables, with significant differences found in the four categories of stumbling in men: frequency of field work, physical burden, low back pain, and frequency of drinking. Significant differences were found in the three categories of falling in men: physical burden, low back pain, and exercise habits. In women, the yes-stumbling group was significantly more likely to have more frequency of field work and have field work. In women, the yes-falling group was significantly more likely to have more frequency of field work [Table 4].

Logistic regression analysis was then conducted to examine independent factors related to the occurrence of stumbling and falling, using perceived stumbling and falling susceptibility as the objective variables. When the odds ratio for a 1 year increase in age was calculated, the result was significant. However, the odds ratio was so close to 1 that it was difficult to interpret, so we calculated the odds ratio for an increase in age of 10 years. In the logistic regression analysis, the Hosmer–Lemeshow test was performed to confirm that the goodness of fit was adequate. Furthermore, the *p*-value of the HL test is shown in the Table 5, Table 6 and Table 7. In addition, because we performed a model selection, we consider that adequate consideration was given to the issue of collinearity. In men, positive associations were found for age (+10 years, odds ratio (OR): 1.98 (95% confidence interval (CI): 1.38–2.84)), mental burden (OR: 2.44 (95% CI: 1.50–3.97)), and frequency of field work (OR: 1.74 (95% CI: 1.09–2.77)), and negative associations for counts of the Seated Stepping Test (OR: 0.95 (95% CI: 0.90–0.99)) and time of TMTB (OR: 0.99 (95% CI: 0.98–1.00)) (Table 5). As for the falling, positive associations were found for age (+10 years, OR: 2.55 (95% CI: 1.65–3.94)), mental burden (OR: 2.40 (95% CI: 1.27–4.53)), exercise habits (OR: 2.55 (95% CI: 1.37–4.76)), and smoking (OR: 2.00 (95% CI: 1.08–3.73)) (Table 6). In women, physical burden was excluded from the initial statistical model because few women answered ‘Strong. Stumbling were significantly related to d TUG (OR: 1.59 (95% CI: 1.06–2.40)) and mental burden (OR: 6.42 (95% CI: 1.75–23.59) (Table 7). In terms of falling, logistic regression was not available due to the small number of outcomes in women. To test for multicollinearity, variance inflation factors (VIFs) were calculated for the variables included after model selection. These low VIFs indicated that the multicollinearity of the obtained model was less likely in the present results.

## 4. Discussion

This study examined the relationship between stumbling and falling in the workplace and physical and cognitive function measurement items. In a comparison of the characteristics of continuous and categorical variables in men between the yes-stumbling group and the no-stumbling group, significant differences were found in 12 items: age, Repeated Rise Test, Closed-Eye One-Leg Test, Seated Stepping Test, TMTA, TMTB, TUG, d_TUG, frequency of field work, physical burden, low back pain, and frequency of drinking. In women, significant differences were found in 4 items: TUG, d_TUG, frequency of field work, and type of occupation. The yes-stumbling group was significantly rated low to the no-stumbling group for all measurement items. In the comparison of the characteristics of the yes-falling and no-falling groups, in men, significant differences were found in 10 items: age, grip strength, Closed-Eye One-Leg Test, Seated Stepping Test, TMTA, TMTB, TUG, physical burden, low back pain, and exercise habits. In women, significant differences were found in d_TUG and frequency of field work. The yes-falling group was also inferior to the no-falling group in all measurement items.

To our knowledge, this is the first report to examine the relationship between stumbling and falling in the workplace and physical and cognitive functions. We also consider that having assessed cognitive function and recognized potential associations in this study, we have taken the first step toward assessing workers’ cognitive function and applying it to accident prevention by further deepening the association with other accident and incident factors in the future. Furthermore, this study selected measurement items that are relatively easy to implement among those used in previous reports on the relationship between physical and cognitive function in the elderly [10,11]. The test items were measured using readily available items, such as paper, pencils, chairs, and grip strength gauges. Therefore, any business establishment does not need much preparation to start the health check, and the measurement methods are easy to implement. Therefore, we believe that it will be relatively easy for small and medium-sized companies without medical specialists to implement the program at the same time as periodical health examinations or at the timing of various health events.

No evaluation system has yet been developed that enables workers alone to easily measure the decline in functions that should be maintained for safe work in the workplace. We will continue to work toward the development of a measurement system that can be implemented in small and medium-sized companies without medical professionals and that can be operated by anyone. We believe it is necessary to further examine the accuracy of this evaluation system and examine its validity by further examining each gender, age, and job category through continued surveys. Furthermore, as reported by Kimura et al. [12], who suggested that maintaining basic physical motor skills can delay age-related memory decline, we would like to prevent occupational accidents by maintaining physical and cognitive functions through training programs that focus on the declining functions obtained from the assessment results.

Schillings et al. [13] studied the stumble response of older adults and compared it with that of younger adults. They reported that older adults were at higher risk of stumbling than younger adults because they had an increased midlatency EMG response and smaller EMG amplitudes in the upper leg muscles than younger adults. Similar results may have been obtained in this study, as both the yes-stumbling and yes-falling groups in men were significantly older. In this study, the Repeated Rise Test scores were significantly lower in the yes-stumbling group in men, suggesting that lower-limb muscle weakness is one of the factors contributing to stumbling. This result is consistent with the fact that the amplitude of the EMG of the upper leg muscles was smaller in the elderly participants than in the younger participants in the aforementioned previous study. In addition, this study showed significantly lower grip strength in the yes-falling group. Ikeda et al. [14] examined the correlation between grip strength and other physical functions in 26 elderly women living in the community. They found significant correlations between grip strength and foot grasp strength, quadriceps muscle strength, skeletal muscle mass, upper body raising, one-leg standing time, 10-m obstacle walking, and 6-min walk tests. In the same way, it is thought that elderly workers with decreased grip strength also have other decreased physical functions, resulting in an increased risk of falls and accidents. In our study, the results of the Closed-Eye One-leg Test were also significantly shorter for both the yes-stumbling group in men and the yes-falling group in men, suggesting that muscle strength and sense of balance could be evaluated as the Closed-Eye One-leg Rest.

Kobayashi et al. [15] compared the results of motor function measurements between two groups of 78 community-dwelling elderly persons (mean age of 78.5 years) according to whether they had fallen in the past year. The same results were obtained in men in this study, suggesting that reduced agility may be one of the factors contributing to falls.

Kitayuguchi et al. [16] examined the association of low back pain and knee pain with stumbles and falls in 491 community-dwelling elderly persons aged 60 years and older. They reported that low back pain was not associated with stumbles but was significantly associated with falls, and knee pain was associated with stumbles and multiple falls, regardless of pain severity. In this study, however, the results differed from previous studies, with significantly more in the yes-stumbling group in men and in the yes-falling group in men having low back pain. In general, the workplace is considered to have more environmental factors for stumbling and falling compared with the living environment. It is possible that factors specific to this type of workplace may be influencing the results.

Alcohol consumption is associated with an increased risk of hospitalization for falls [17]. A study in 158 older adults also reported that exercise affects the maintenance of physical, cognitive, and mental function in the elderly [6]. Our study also found an association between drinking and exercise habits and stumbling and falling, supporting previous reports.

Oya et al. [18] examined the relationship between stride size and stumbling in 68 community-dwelling elders and reported that TUG was a significant factor influencing stumbling. In this study, TUG was significantly inferior in the yes-stumbling group in men and women, and in the yes-falling group in men, the mean value was inferior, which seems to confirm the previous study. It is also considered to be a simple and useful measurement method. Soubra et al. [10] emphasized that TUG is a highly recommended measure because it includes simple activities of daily living and daily tasks (standing, walking, turning).

Shumway-Cook et al. [19] reported that TUG, d_TUG with a subtraction task, and d_TUG while carrying a full glass of water were administered to 30 community-dwelling older adults and compared 15 subjects in the group of elders with a history of falls and 15 subjects in the group of elders without a history of falls, suggesting that TUG is a highly sensitive (87%) and specific (87%) index for identifying community-dwelling adults at risk of falls. Langeard et al. [20] also measured cognitive and walking performance in 65 subjects under single-task and dual-task conditions. They reported that the dual task was useful in detecting cognitive and motor decline because of its greater sensitivity to age. Since a report by Zijlstra et al. [21] indicated that the dual task may have added value in predicting falls, we also selected TUG with d_TUG as a measurement item in this study. Significant results were obtained for the yes-stumbling group in both men and women and the yes-falling group in men, suggesting that the dual task may be associated with stumbling and falling, as in previous studies.

However, not all items that showed significant correlations in these univariate analyses also showed significant odds ratios in the logistic regression analysis. The five items that showed significant differences in both univariate and logistic regression analysis were age, Seated Stepping Test, TMTB, frequency of field work, and mental burden for stumbling in men, only d_TUG for stumbling in women and two items for falling in men, age and exercise habits. These items also track the results of previous studies, as noted above, and are considered possible associations with stumbling and falling. On the other hand, the items that showed significant differences in univariate analysis but not in logistic regression analysis were: for stumbling in men, eight items were the Repeated Rise Test, Closed-Eye One-Leg Test, TMTA, TUG, d_TUG, physical burden, low back pain, and frequency of drinking; for stumbling in women, three items were TUG, frequency of field work, and type of occupation; for falling in men, eight items were grip strength, Closed-Eye One-Leg Test, Seated Stepping Test, TMTA, TMTB, TUG, physical burden, and low back pain. We will continue to investigate these items further to clarify the association with stumbling and falling.

The strengths of this study include its reference to the cognitive function of older adults in the workplace and its use of simple measurement items that can be administered in settings without the assistance of medical professionals. On the other hand, the study had the following limitations. The lack of a medical history and a current medical history in the questionnaire item and its impact on the measurement results was not examined. The lack of years of work in the questionnaire item and its impact on the measurement results was also not examined. In addition, the impact of differences in the type of occupation of the participants was not determined. Another limitation is the overlapping of a certain number of risks, as some participants in this study reported experiencing both “stumbling” and “falling”. To address these limitations, we consider that it is necessary to continue the survey and add more questionnaire items and also expand it to include occupations with a low number of participants. Furthermore, while we have taken the first step toward assessing workers’ cognitive function, consideration should be given to adding more cognitive function tests in the future to further clarify the impact of cognitive function.

## 5. Conclusions

Based on the above results, we conclude that a worse performance in the test performed may have an impact on stumbling and falling in elderly workers. This result is consistent with previous studies on community-dwelling elderly people and home-disabled elderly people. We consider that this study demonstrated the usefulness of a system for evaluating physical and cognitive functions in a simple and convenient manner using only workers. The findings of this study support the possibility of establishing such a system and applying it to accident prevention and health promotion. We plan to continue to develop and establish systems for evaluating physical and cognitive functions.

## Figures and Tables

**Table 1:** Questionnaire and classification.

Questionnaire	Classification
Frequency of Field Work: “How often do you work in the field (including patrol, observation, and guidance)?”	
Rarely	Low
One day a week	Low
Two days a week	Low
Three days a week	High
Four or more days a week	High
Physical Burden: “How much do you think the physical burden of your job is?”	
Weak	Weak
Normal	Weak
Strong	Strong
Fairly strong	Strong
Mental Burden: “How much do you think the mental burden of your job is?”	
Weak	Weak
Normal	Weak
Strong	Strong
Fairly strong	Strong
Low Back Pain: “Do you have low back pain?”	
No	No
Sometimes	No
Often	Yes
Always	Yes
Knee Pain: “Do you have knee pain?”	
No	No
Sometimes	No
Often	Yes
Always	Yes
Exercise Habits: “Do you exercise habitually (the guideline is at least 2 days per week for at least 30 min at a time)?”	
Yes	Yes
Occasionally	Yes
No	No
Smoking: “Do you smoke?”	
No	No
1–10 cigarettes per day	Yes
11–20 cigarettes per day	Yes
21–40 cigarettes per day	Yes
41 or more cigarettes per day	Yes
Frequency of Drinking: “How many days a week do you drink alcohol?”	
0	Low
1 day	Low
2 days	Low
3 days	Low
4 days	Low
5 days	High
6 days	High
7 days	High
Type of Occupation	
Manufacturing: jobs at manufacturing sites for automobiles, steel, chemical products, food, clothing	Field work
Construction: construction workers, civil engineering workers, scaffolders, carpenters, electricians, plasterers	Field work
Transportation and driving: truck drivers, cabdrivers, train drivers	Field work
Delivery and cleaning: delivery persons, cleaners	Field work
Agriculture and fishing: agriculture, fishing, livestock farming	Field work
Security: security guards, police officers	Field work
Service: cooks, salespeople	Nonfield work
Office work: general office work, accounting work, sales clerks	Nonfield work
Professional work: medical specialists, researchers, engineers	Nonfield work
Stumbling: “Do you feel likely to stumble easily on flat surfaces and small bumps at work?”	
No	No-stumbling
Yes	Yes-stumbling
Falling: “At work, do you feel likely to fall or wobble on stairs, ladders, stepladders, etc.?”	
No	No-falling
Yes	Yes-falling
Getting stuck or entangled: “Do you often get caught, stuck, or entangled in machinery while working?”	
No	No-getting stuck or entangled
Yes	Yes-getting stuck or entangled

**Table 2:** Distribution of outcome grouped according to sex.

	Men			Women		
	Total	No	Yes	Total	No	Yes
	*n*	*n* (%)	*n* (%)	*n*	*n* (%)	*n* (%)
Stumbling	605	403 (66.6%)	202 (33.4%)	119	72 (60.5%)	47 (39.5%)
Falling	605	521 (86.1%)	84 (13.9%)	119	104 (87.4%)	15 (12.6%)
Getting stuck or entangled	605	599 (99.0%)	6 (1.0%)	118	118 (100.0%)	0 (0.0%)
Stumbling and falling	605	539 (89.1%)	66 (10.9%)	119	105 (88.2%)	14 (11.8%)

**Table 3:** Characteristics of continuous variables grouped according to the outcomes and sex.

	Total		Stumbling			Falling		
			No	Yes		No	Yes	
	*n*	Mean (SD)	Mean (SD)	Mean (SD)	*p*	Mean (SD)	Mean (SD)	*p*
Men								
Age (years)	605	53.3 (7.5)	52.5 (7.4)	55.1 (7.3)	<0.001	52.9 (7.4)	56.3 (7.6)	<0.001
Grip strength (kg)	604	41.5 (6.1)	41.7 (6.2)	41.1 (5.9)	0.381	41.7 (6.1)	39.8 (6.1)	0.006
Repeated Rise Test (times/30 s)	568	23.6 (6.2)	24.1 (6.1)	22.6 (6.3)	0.003	23.7 (6.1)	22.8 (6.8)	0.122
Closed-Eye One-Leg Test (s)	600	20.5 (21.8)	22.8 (23.4)	15.9 (17.2)	<0.001	21.4 (22.6)	14.8 (14.9)	0.017
FRT (cm)	599	38.2 (6.7)	38.4 (6.4)	37.7 (7.3)	0.239	38.3 (6.8)	37.4 (6.4)	0.143
Seated Stepping Test (times/20 s)	592	34.4 (6.2)	35.1 (6.0)	32.9 (6.4)	<0.001	34.7 (6.1)	32.3 (6.7)	0.002
TMTA (s)	602	86.3 (28.1)	84.6 (28.5)	89.7 (27.1)	0.002	85.0 (27.1)	94.5 (32.4)	0.004
TMTB (s)	604	97.8 (35.9)	96.1 (35.1)	101.4 (37.2)	0.040	96.8 (36.3)	104.5 (33.0)	0.004
TUG (s)	469	5.8 (0.9)	5.7 (0.8)	6.0 (0.9)	<0.001	5.8 (0.9)	6.0 (0.8)	0.023
d_TUG (s)	469	8.6 (5.4)	8.5 (6.5)	8.8 (1.8)	<0.001	8.5 (5.8)	8.7 (1.9)	0.051
Women								
Age (years)	119	52.3 (7.7)	52.4 (7.5)	52.2 (8.2)	0.613	51.8 (7.4)	56.0 (9.0)	0.087
Grip strength (kg)	119	25.1 (4.5)	24.6 (4.5)	25.9 (4.4)	0.128	25.0 (4.5)	25.5 (4.6)	0.692
Repeated Rise Test (times/30 s)	88	24.9 (6.2)	25.5 (6.1)	24.0 (6.3)	0.198	24.8 (5.9)	25.2 (8.7)	0.880
Closed-eye One-Leg Test (s)	118	19.2 (20.2)	19.5 (19.4)	18.8 (21.5)	0.449	19.7 (20.4)	16.0 (19.0)	0.304
FRT (cm)	118	37.5 (5.9)	37.2 (5.7)	38.0 (6.3)	0.195	37.3 (6.1)	38.9 (3.9)	0.258
Seated Stepping Test (times/20 s)	118	34.3 (5.0)	34.5 (5.0)	34.0 (5.1)	0.799	34.4 (5.2)	33.6 (3.6)	0.799
TMTA (s)	119	79.0 (19.5)	80.4 (20.5)	76.8 (17.8)	0.445	79.2 (20.2)	77.2 (14.2)	0.835
TMTB (s)	119	88.0 (31.4)	89.8 (34.8)	85.3 (25.3)	0.628	87.5 (32.5)	91.5 (22.4)	0.189
TUG (s)	75	6.0 (0.9)	5.8 (0.9)	6.3 (0.9)	0.014	6.0 (0.9)	6.3 (0.9)	0.349
d_TUG (s)	75	8.2 (1.7)	7.8 (1.5)	8.8 (1.9)	0.017	8.0 (1.6)	10.0 (1.4)	0.005

SD: Standard deviation; *p*: *p*-values by Mann–Whitney U test.

**Table 4:** Distribution of characteristics of categorical variables grouped according to the outcomes.

		Total	Stumbling			Falling		
			No	Yes		No	Yes	
		*n* (%)	*n* (%)	*n* (%)	*p*	*n* (%)	*n* (%)	*p*
Men								
Frequency of field Work	High	310 (51.2%)	193 (47.9%)	117 (57.9%)	0.025	262 (50.3%)	48 (57.1%)	0.290
Physical burden	Strong	83 (13.7%)	44 (10.9%)	39 (19.3%)	0.006	64 (12.3%)	19 (22.6%)	0.016
Mental burden	Strong	241 (39.8%)	153 (38.0%)	88 (43.6%)	0.188	202 (38.8%)	39 (46.4%)	0.189
Low back pain	Yes	121 (21.3%)	72 (19.1%)	49 (25.4%)	<0.001	96 (19.7%)	25 (30.9%)	0.028
Knee pain	Yes	67 (11.8%)	31 (8.2%)	36 (18.7%)	0.104	53 (10.9%)	14 (17.3%)	0.134
Exercise habits	No	272 (45.0%)	176 (43.7%)	96 (47.5%)	0.387	221 (42.4%)	51 (60.7%)	0.002
Smoking	Yes	209 (34.5%)	139 (34.5%)	70 (34.7%)	1.000	176 (33.8%)	33 (39.3%)	0.325
Frequency of drinking	High	269 (44.5%)	161 (40.0%)	108 (53.5%)	0.002	228 (43.8%)	41 (48.8%)	0.409
Type of occupation	Nonfield work	291 (48.1%)	202 (50.1%)	89 (44.1%)	0.168	251 (48.2%)	40 (47.6%)	1.000
Women								
Frequency of field Work	High	20 (19.6%)	8 (12.9%)	12 (30.0%)	0.043	15 (16.5%)	5 (45.5%)	0.037
Physical burden	Strong	4 (3.9%)	1 (1.6%)	3 (7.5%)	0.297	3 (3.3%)	1 (9.1%)	0.371
Mental burden	Strong	34 (33.3%)	16 (25.8%)	18 (45.0%)	0.055	29 (31.9%)	5 (45.5%)	0.499
Low back pain	Yes	10 (11.5%)	4 (7.8%)	6 (16.7%)	0.307	8 (10.5%)	2 (18.2%)	0.608
Knee pain	Yes	10 (11.5%)	6 (11.8%)	4 (11.1%)	1.000	8 (10.5%)	2 (18.2%)	0.608
Exercise habits	No	60 (50.4%)	34 (47.2%)	26 (55.3%)	0.455	51 (49.0%)	9 (60.0%)	0.582
Smoking	Yes	43 (36.1%)	27 (37.5%)	16 (34.0%)	0.845	36 (34.6%)	7 (46.7%)	0.397
Frequency of drinking	High	15 (12.6%)	12 (16.7%)	3 (6.4%)	0.156	14 (13.5%)	1 (6.7%)	0.690
Type of occupation	Nonfield work	109 (91.6%)	69 (95.8%)	40 (85.1%)	0.049	97 (93.3%)	12 (80.0%)	0.113

*p*: *p*-values by χ-square test.

**Table 5:** Odds ratios and 95% confidence intervals for stumbling after and before model selection in men.

	OR (95% CI)	B	Wald	*p*	VIF
After model selection * (*p*-value of Hosmer–Lemeshow test: 0.927)					
Age (+10 years)	1.98 (1.38, 2.84)	0.682	13.760	<0.001	1.351
Seated Stepping Test (+1 times/20 s)	0.95 (0.90, 0.99)	0.056	6.496	0.011	1.267
TMTB (+1 s)	0.99 (0.98, 1.00)	0.008	4.123	0.042	1.438
TUG (+1 s)	1.32 (0.98, 1.78)	0.275	3.227	0.072	1.238
Frequency of field work (high/low)	1.74 (1.09, 2.77)	0.553	5.440	0.020	1.052
Mental burden (strong/weak)	2.44 (1.50, 3.97)	0.892	12.885	<0.001	1.093
Knee pain (yes/no)	2.25 (0.99, 5.10)	0.811	3.788	0.052	1.058
Before model selection					
Age (+10 years)	1.86 (1.24, 2.80)	0.623	9.017	0.003	
Grip strength (+1 kg)	1.03 (0.98, 1.08)	0.031	1.725	0.189	
Repeated Rise Test (+1 times/30 s)	1.01 (0.97, 1.05)	0.009	0.156	0.693	
Closed-Eye One-Leg Test (+1 s)	0.99 (0.98, 1.01)	0.008	1.506	0.220	
FRT (+1 cm)	1.00 (0.96, 1.04)	0.002	0.007	0.936	
Seated Stepping Test (+1 times/20 s)	0.94 (0.89, 0.99)	0.062	5.578	0.018	
TMTA (+1 s)	1.01 (0.99, 1.02)	0.005	0.990	0.320	
TMTB (+1 s)	0.99 (0.98, 1.00)	0.010	4.575	0.032	
TUG (+1 s)	1.39 (1.01, 1.91)	0.332	4.194	0.041	
d_TUG (+1 s)	0.98 (0.93, 1.04)	0.017	0.395	0.530	
Frequency of field work (high/low)	1.76 (1.03, 3.00)	0.565	4.330	0.037	
Physical burden (strong/weak)	1.71 (0.78, 3.75)	0.538	1.819	0.177	
Mental burden (strong/weak)	2.28 (1.37, 3.80)	0.824	10.030	0.002	
Low back pain (yes/no)	1.14 (0.61, 2.12)	0.129	0.165	0.685	
Knee Pain (yes/no)	2.52 (1.06, 6.01)	0.926	4.372	0.037	
Exercise habits (no/yes)	1.27 (0.79, 2.05)	0.238	0.952	0.329	
Smoking (yes/no)	0.86 (0.50, 1.47)	0.151	0.306	0.580	
Frequency of drinking (high/low)	1.28 (0.80, 2.06)	0.250	1.080	0.299	
Type of occupation (nonfield/field)	1.41 (0.79, 2.51)	0.342	1.344	0.246	

OR: Odds ratios, 95% CI: 95% confidence intervals. B: beta coefficient. Wald: Wald statistic. VIF: variance inflation factor. * Indicated covariates were selected using backward elimination methods.

**Table 6:** Odds ratios and 95% confidence intervals for falling after and before model selection in men.

	OR (95% CI)	B	Wald	*p*	VIF
After model selection * (*p*-value of Hosmer–Lemeshow test: 0.092)					
Age (+10 years)	2.55 (1.65, 3.94)	0.938	17.897	<0.001	1.046
Mental burden (strong/weak)	2.40 (1.27, 4.53)	0.877	7.341	0.007	1.047
Exercise habits (no/yes)	2.55 (1.37, 4.76)	0.937	8.688	0.003	1.001
Smoking (yes/no)	2.00 (1.08, 3.72)	0.695	4.832	0.028	1.001
Before model selection					
Age (+10 years)	2.83 (1.60, 5.01)	1.040	12.716	<0.001	
Grip strength (+1 kg)	0.96 (0.90, 1.02)	0.045	1.862	0.172	
Repeated Rise Test (+1 times/30 s)t	1.06 (0.99, 1.12)	0.055	3.059	0.080	
Closed-Eye One-Leg Test (+1 s)	1.00 (0.98, 1.02)	0.001	0.011	0.916	
FRT (+1 cm)	0.99 (0.94, 1.05)	0.006	0.045	0.831	
Seated Stepping Test (+1 times/20 s)	0.96 (0.89, 1.03)	0.042	1.336	0.248	
TMTA (+1 s)	1.00 (0.99, 1.01)	0.001	0.027	0.869	
TMTB (+1 s)	0.99 (0.98, 1.00)	0.010	2.887	0.089	
TUG (+1 s)	1.33 (0.85, 2.06)	0.283	1.581	0.209	
d_TUG (+1 s)	1.00 (0.93, 1.09)	0.004	0.011	0.918	
Frequency of field work (high/low)	1.52 (0.73, 3.14)	0.416	1.247	0.264	
Physical burden (strong/weak)	1.34 (0.45, 4.02)	0.295	0.278	0.598	
Mental burden (strong/weak)	2.29 (1.13, 4.64)	0.828	5.270	0.022	
Low back pain (yes/no)	0.97 (0.42, 2.29)	−0.026	0.004	0.952	
Knee pain (yes/no)	1.43 (0.45, 4.53)	0.361	0.377	0.539	
Exercise habits (no/yes)	2.80 (1.43, 5.50)	1.031	9.010	0.003	
Smoking (yes/no)	2.10 (1.05, 4.18)	0.740	4.412	0.036	
Frequency of drinking (high/low)	1.11 (0.58, 2.14)	0.108	0.106	0.745	
Type of occupation (nonfield/field)	1.49 (0.67, 3.31)	0.398	0.954	0.329	

OR: Odds ratios, 95% CI: 95% confidence intervals. B: beta coefficient. Wald: Wald statistic. VIF: variance inflation factor. * Indicated covariates were selected using backward elimination methods.

**Table 7:** Odds ratios and 95% confidence intervals for stumbling after and before model selection in women.

	OR (95% CI)	B	Wald	*p*	VIF
After model selection * (*p*-value of Hosmer–Lemeshow test: 0.641)					
Age (+10 years)	0.35 (0.12, 1.07)	−1.040	3.394	0.065	1.016
d_TUG (+1 s)	1.59 (1.06, 2.40)	0.466	4.970	0.026	1.009
Mental burden (strong/weak)	6.42 (1.75, 23.59)	1.860	7.847	0.005	1.012
Before model selection					
Age (+10 years)	0.40 (0.07, 2.37)	−0.918	1.022	0.312	
Grip strength (+1 kg)	1.12 (0.94, 1.35)	0.116	1.570	0.210	
Repeated Rise Test (+1 times/30 s)	0.90 (0.76, 1.08)	−0.100	1.210	0.271	
Closed-Eye One-Leg Test (+1 s)	1.00 (0.97, 1.04)	0.003	0.024	0.878	
FRT (+1 cm)	1.05 (0.91, 1.23)	0.053	0.486	0.486	
Seated Stepping Test (+1 times/20 s)	1.03 (0.85, 1.26)	0.031	0.096	0.757	
TMTA (+1 s)	1.00 (0.95, 1.06)	0.003	0.012	0.911	
TMTB (+1 s)	0.99 (0.95, 1.02)	−0.014	0.602	0.438	
TUG (+1 s)	2.58 (0.77, 8.61)	0.948	2.379	0.123	
d_TUG (+1 s)	1.38 (0.69, 2.77)	0.321	0.814	0.367	
Frequency of field work (high/low)	4.74 (0.27, 82.97)	1.556	1.135	0.287	
Mental burden (strong/weak)	9.11 (1.35, 61.40)	2.209	5.147	0.023	
Low back pain (yes/no)	8.31 (0.14, 490.80)	2.117	1.035	0.309	
Knee pain (yes/no)	0.10 (0.00, 6.84)	−2.341	1.158	0.282	
Exercise habits (no/yes)	1.39 (0.26, 7.42)	0.330	0.149	0.699	
Smoking (yes/no)	0.22 (0.01, 3.43)	−1.510	1.165	0.280	
Frequency of drinking (high/low)	0.49 (0.05, 5.35)	−0.704	0.336	0.562	
Type of occupation (nonfield/field)	0.21 (0.01, 5.91)	−1.562	0.841	0.359	

OR: Odds ratios, 95% CI: 95% confidence intervals. B: beta coefficient. Wald: Wald statistic. VIF: variance inflation factor. * Indicated covariates were selected using backward elimination methods.

## Data Availability

The data that support the findings of this study are available from the corresponding authors upon reasonable request.
